# Micro-CT analysis of dentinal microcracks on root canals filled with a bioceramic sealer and retreated with reciprocating instruments

**DOI:** 10.1038/s41598-020-71989-6

**Published:** 2020-09-17

**Authors:** Andressa Almeida, Kaline Romeiro, Marcely Cassimiro, Luciana Gominho, Eugênia Dantas, Silmara Silva, Diana Albuquerque

**Affiliations:** 1 Department of Operative Dentistry and Endodontics, Dental College of Pernambuco, University of Pernambuco (UPE), Avenida General Newton Cavalcanti, 1650, Camaragibe, PE 54753-020 Brazil; 2 Department of Clinic and Social Dentistry, Federal University of Paraiba (UFPB), João Pessoa, PB Brazil

**Keywords:** Health care, Risk factors

## Abstract

This study aimed to analyze the potential occurrence of dentinal defects after the removal of a root canal filling with two different sealers using Reciproc (RC) or Reciproc Blue (RB). The mesial roots of 60 mandibular molars with a Vertucci type IV configuration were selected. The samples were initially instrumented with Reciproc (R25) and then divided into the following four experimental groups according to the endodontic sealer and retreatment instrument (n = 15): BC Sealer/Reciproc (BCRC); BC Sealer/Reciproc Blue (BCRB); AH Plus/Reciproc (AHRC); and AH Plus/Reciproc Blue (AHRB). Then, the samples were scanned under micro-CT after obturation and removal of the filling material. Two analyses were conducted. First, an evaluation was performed on all the axial images, and another analysis evaluated each millimeter of the 10 mm from the apex. Dentinal defects were observed in all the samples. All of the identified defects in the images after filling material removal were present in the corresponding images after obturation. The use of AH Plus and EndoSequence BC Sealer, and filling material removal using RC and RB instruments did not induce dentinal defects.

## Introduction

Retreatment procedures involve the removal of the previous filling material to allow thorough reinstrumentation, disinfection, and refilling of the root canal system^1^. The resistance imposed by the filling material can make retreatment a stressful and time-consuming procedure, especially in curved canals^2^. In retreatment, as more dentin is removed to remodel and disinfect the root canal, defects are likely to arise^3^. This formation has been questioned due to different evaluation methods^4–9^. Microcomputed tomography is the gold standard method in some in vitro studies in endodontics and can be used with accuracy to assess dentinal defects^7–10^.

The instrument used in retreatment may influence the occurrence of dentinal defects^11^. Several endodontic instruments have been evaluated for filling material removal of the root canal systems^12–14^. Reciprocating instruments are well established in the literature for root canal filling material removal. The Reciproc (VDW, Munich, Germany) and Reciproc Blue (VDW, Munich, Germany) have been shown to be effective in removing filling materials from root canals filled with resin cements^9,15–19^.

Different bioceramic sealers have recently been proposed and are currently being studied. These sealers are characterized by bioactivity and the ability to form hydroxyapatite during the curing process. Thus, they have the ability to chemically bond to root dentin^20^. Whether the formation of hydroxyapatite at the dentinal-material interface strengthens the root’s structure has not be established. The EndoSequence BC bioceramic sealer (Brasseler USA, Savannah, GA) has shown superior bond strength compared to other sealers^21^; however, in the case of retreatment, the use of bioceramics is more complex to remove the filling material^22,23^. Whether dentinal defects form due to the unblocking of obturated canals with bioceramic cement is unknown^22,23^.

The present study aimed to evaluate the occurrence of dentinal defects after filling material removal y using Reciproc or Reciproc Blue files on the mesial roots of mandibular molars that were obturated with either AH Plus or EndoSequence BC sealers and gutta-percha. The null hypothesis is that the retreatment procedures do not generate dentinal defects.

## Methods and materials

This research was approved by the Institutional Research Ethics Committee (Protocol: 65455816.8.0000.5207).

### Sample size calculation

The sample size of each group was calculated according to Coelho et al.^24^. Statistical software (Epi Info™ 6 for Windows; Centers for Disease Control and Prevention, Georgia, USA) was used with a 5% margin of error, and a sample size with a power of 80% would consist of 15 molars.

### Sample selection

Initially, 328 first and second human mandibular molars with a Vertucci type IV configuration were selected^25^. The reasons for extraction of these teeth were unrelated because they were obtained from a Human Tooth Bank (HTB). All the teeth were disinfected in a 0.1% thymol solution over 24 h, kept in purified filtered water for 30 days, and then underwent micro-CT analysis. The molars were examined under stereomicroscopy (Labomed Luxeo 4D, Los Angeles, CA, USA) (15× magnification) to analyze the existence of external cracks. The curvature angles were chosen based on the initial radiographs using ImageJ software (Version 1.46r, National Institutes of Health, Bethesda, MD). Only the teeth with a moderate curvature of the mesial root (10°–20°)^26^ and more than 17 mm in length with fully formed apices were selected. After radiographic analysis, the teeth that presented previous endodontic treatment, pulp calcification, root resorption and 2 mesial canals with the same apical foramen were excluded. Sixty teeth were selected according to these criteria. Only one operator performed the root canal preparation, obturation and filling removal procedures.

### Sample preparation

The coronal portions were removed using a double-sided diamond disk under refrigeration to standardize the samples with ± 17 mm length. Then, the teeth were accessed, and the glide path was established by inserting a size #10 stainless steel K-file (Dentsply Maillefer, Ballaigues, Switzerland). The working length (WL) was determined 1 mm short from the apical foramen. A thin layer of light body addition silicone was used to simulate the periodontal ligament in acrylic resin blocks. Both mesial root canals were prepared with a Reciproc instrument R25 (25/0.08v). The file was then introduced into the root canal until resistance was felt, and 3 forward–backward movements were performed with slight apical pressure. The instrument was removed from the canal and cleaned. The irrigation was performed with 1 mL of 2.5% sodium hypochlorite (NaOCl) after each insertion of the file. An irrigation with 2 mL of 17% EDTA was performed to remove the smear layer, and a final irrigation was performed with 2 mL of 2.5% NaOCl. The samples were randomly distributed into the following 4 experimental groups according to the endodontic sealer and the instrument that was used (https://www.random.org) (n = 15): (1) BCRC—EndoSequence BC Sealer/Reciproc; (2) BCRB—EndoSequence BC Sealer/Reciproc Blue; (3) AHRC—AH Plus/Reciproc; and (4) AHRB—AH Plus/Reciproc Blue.

### Root canal obturation

All samples were filled through the single cone technique^27^ using a Reciproc System R25 gutta-percha cone (VDW, Munich, Germany) adjusted on WL ± 16 mm. The canals were dried with Reciproc System R25 paper points (VDW, Munich, Germany). Two groups (n = 30) were filled with AH Plus Sealer, and the other groups (n = 30) were filled with EndoSequence BC Sealer*.* The AH Plus was manipulated according to the manufacturer’s instructions. This sealer was carried onto the tip of a Lentulo spiral (Dentsply Sirona Endodontics, Ballaigues, Switzerland) that advanced slowly until 1 mm short from the WL running at low speed. After the cone/sealer insertion, the gutta-percha was cut through its cervical portion and compacted vertically to the cementoenamel junction with a heated instrument. The filling procedure using EndoSequence BC Sealer was performed by positioning the syringe tip inside the root canal and injecting the sealer according to the manufacturer’s instructions. Then, the gutta-percha was inserted into the WL. The gutta-percha cone was cut and compacted similarly to the previous group. Radiographs were taken in both the buccolingual and mesiodistal directions to assess the quality of the filling procedure. The crowns were sealed with a temporary filling material (Cavit; 3M ESPE, St Paul, MN) and stored at 37 °C at 100% humidity for 30 days^13^.

### Micro-CT analysis

Scanning was performed individually under micro-CT (SKYSCAN 1172, Bruker, Belgium). Each tooth was positioned on the turntable with the roots facing upwards. The parameters used were as follows: 100 kV voltage and a 100 μA current, with a resolution of 17.87 μm; 360° rotation using a 1 mm thick copper + aluminum filter and a 0.5 rotation step, under 34 min of exposure. After scanning, the images were reconstructed using the software NRecon (SkyScan, Kontich, Belgium), producing 700–800 images per tooth.

### Retreatment procedures

The root filling materials were removed from both mesial root canals using either Reciproc R40 or Reciproc Blue RB40 instruments (n = 15) in reciprocating motion with a VDW Silver motor in its recommended settings for such files. The R40 and RB40 files were introduced into the root canal in 3 forward–backward movements with 3 mm of amplitude with slight apical pressure. After those movements, the file was removed from the root canal and cleaned using sterilized gauzes. The canals were irrigated with approximately 2 mL of NaOCl before reinserting the file. The instrument was then used until the WL was reached. The filling removal procedure was finished when the file ceased to present gutta-percha residues. Finally, irrigation was performed with 2 mL of 17% EDTA to remove the smear layer, followed by a final irrigation using 2 mL of 2.5% NaOCl. The total NaOCl volume used was 12 mL. After the filling removal procedures were completed, the sample was scanned again under micro-CT using the same parameters that were previously described.

### Dentinal defects evaluation

Initially, the software DataViewer (SkyScan, Kontich, Belgium) was used for coregistration of the two sets of images to align them geographically, thereby generating an overlapping image in the same position. After this step, the images were analyzed using the software CTAn (SkyScan, Kontich, Belgium). The region of interest (ROI) selected for each sample was the apical 10 mm of the mesial root.

Based on divergent results reported in previous studies, the present research used two different methods to evaluate the micro-CT images as proposed by Cassimiro et al*.*^28^. Both analyses were performed only on the apical 10 mm of the mesial root. In the first analysis, all of the images from this region were screened to identify and account for the presence of dentinal defects^29,30^*.* The second analysis evaluated only one image in 1 mm intervals^31^.

A dentinal defect was binomially categorized as “no defect = no” and “defect = yes”^31^. If no dentinal defects or craze lines were present on the external surface of the root or on the internal root canal wall, the slice was labeled “no defect”. If there were any craze lines, dentinal defects or fractures in the root dentine, the slice was labeled ‘defect’.

In both analyses, the images of the desobturated teeth were observed and images with dental defects were cataloged. Subsequently, the cataloged images were compared to the corresponding images of the obturated teeth.

To validate the process, both analyses were repeated after 2 weeks. In the case of divergence, the images were analyzed at the same time by three evaluators until a consensus was reached.

## Results

**Figure 1:**
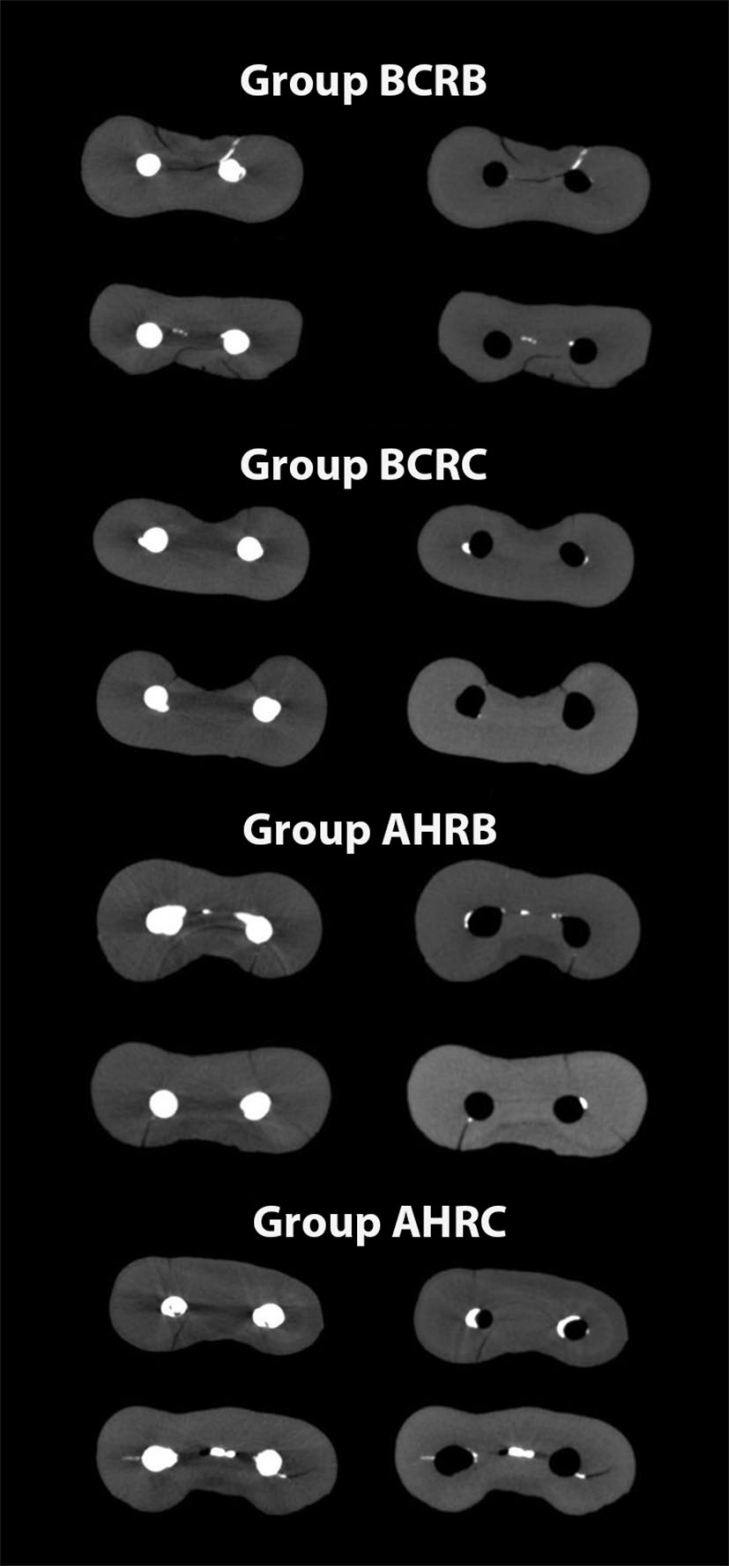
Representative micro-CT cross-section images of the mandibular mesial root, 5 mm from the apical foramen, after obturation and filling removal procedures for the BCRB, BCRC, AHRB and AHRC groups.

All the samples presented pre-existing dentinal defects. All the identified dentinal defects after the removal of the filling material were also present in the corresponding images after obturation. Therefore, no new dentinal defects were formed (Fig. 1).

The first analysis evaluated a total of 43,200 images from all samples, and 24.27% (10,486 images) presented dentinal defects after root canal filling and retreatment. Of those, 20.06% (2,104 images) were observed in the BCRB group, 28.64% (3,003 images) in the BCRC group, 34.11% (3,577 images) in the AHRB group and 17.19% (1802 images) in the AHRC group.

In the second analysis, a total of 600 images were evaluated. From them, 27.5% (165 images) showed dentinal defects. The defects were observed in 27.88% (46 images), 27.27% (45 images), 33.34% (55 images) and 11.51% (19 images) of the images in the BCRB, BCRC, AHRB and AHRC groups, respectively.

## Discussion

In this study, the null hypothesis was accepted, and the retreatment procedures did not generate new dentinal defects. The results contribute to the consensus regarding the presence of preexisting dentinal defects^14,28–32^. Nevertheless, in preparation of mandibular teeth cadavers, De Deus et al*.*^33^ observed pre-existing microcracks. More recently, another study by De Deus et al*.*^34^ also aimed to investigate the occurrence of preexisting dentinal defects in non-endodontically treated teeth in fresh cadavers. It was suggested that pre-existing dental defects are treated are experimental and formed by storage conditions and/or due to extraction procedures. In our study, the preexisting dentinal defects identified can be justified as experimental due to the samples having been obtained through a tooth bank, from which the causes and the extraction methods are not reported.

Different methods exist for evaluating the formation of dentinal defects following root canal interventions, such as analysis under stereomicroscopy, LED transillumination and more recently micro-CT^6,24,30^. With the technological advances in the field of imaging, the use of micro-CT is already considered the gold standard method to evaluate dentinal defects. This method allows each sample to be its own control after micrometric three-dimensional volumetric reconstruction and therefore a comparison can be made for the same tooth^35^. New dentinal defect formation observed after endodontic procedures in micro-CT images presented by Pop et al*.*^36^, Ceyhanli et al*.*^31^ and Kirici et al.^9^ disagree with most of the results obtained in similar studies^28–30,32,37^. According to De Deus et al.^38^ this contradiction was probably caused by the use of different scanning methods and dentinal defect analyses. An important step in comparing the same sample in two different moments is the performance of the coregistration, known as image overlapping, which creates files with the sample images in identical positions so that they can be analyzed and compared faithfully. Both Pop et al*.*^36^ and Ceyhanli et al.^31^ did not report performing this procedure.

Cassimiro et al*.*^28^ performed the two different methods proposed by Ceyhanli et al*.*^31^ and De Deus et al*.*^30,33,37^ to evaluate dentinal defects using image overlapping and did not obtain different answers regarding to defect formation before and after root preparation, which corroborates the findings of the present research.

Considering the retreatment procedures, filling material removal was related to the presence or propagation of structural defects in the dentin, such as dentinal defects or fractures, in studies with stereomicroscope analysis^4^. Yilmaz et al.^14^ and Koçak et al.^7^ also evaluated the formation of dentinal defects after filling removal procedures in micro-CT. This procedure did not directly influence the formation of dentinal defects even with a final instrument tip size 40 used by Yilmaz et al.^14^ and in the present study.

Both endodontic instruments used in this study had the same design and kinematics, with the only difference being the manufacturing process. The new Blue surface heat treatment of Reciproc Blue has demonstrated superior results, such as better flexibility, compared to the mechanical properties of the Reciproc System instruments^39^. Even so, the results of this research showed no difference regarding the formation of dentinal defects after the use of both files to remove filling material with either AH Plus or EndoSequence BC sealers and gutta-percha. This result demonstrates that the flexibility of the instrument is not a factor that interferes with the formation of dentinal defects. Other studies have also shown that different heat treatments used to produce files did not influence the formation of dentinal defects in endodontics treatments^28,30,32^.

As reported in the literature, when analyzing the filling material in the presence of EndoSequence BC Sealer, this sealer has the potential to form hydroxyapatite crystals at the dentin and cement interface^40^ and presents the same sealing capacity of epoxy-based cements^41^. These properties probably did not influence the results of the dentinal defects in this study, which is corroborated by the findings reported by Ersahan and Aydin^42^ and Zhang et al.^41^.

Regardless of the file used to remove the filling material, patency in the canals filled with EndoSequence BC Sealer was difficult to obtain, but did not influence the formation of dentinal defects. The same difficulty has been reported in other studies^22,23^. and may be due to its self-adhesive nature, which forms a chemical bond with the dentin through the production of hydroxyapatite during the curing process^21^.

## Conclusion

The mesial mandibular molars samples in this study contained pre-existing dentinal defects, but their formation was not observed after filling material removal of the canals that were subsequently filled with AH Plus and EndoSequence BC Sealer and retreated with Reciproc and Reciproc Blue.
